# Nitrogen Biobank for Cardiovascular Research

**DOI:** 10.2174/1573403X113099990035

**Published:** 2013-08

**Authors:** Antonella Mercuri, Stefano Turchi, Andrea Borghini, Maria Rosa Chiesa, Guido Lazzerini, Laura Musacchio, Ottavio Zirilli, Maria Grazia Andreassi

**Affiliations:** 1 U.O. Biobank, CNR, Institute of Clinical Physiology, Pisa, Italy;; 2 Air Liquide Sanità Service, Milano, Italy

**Keywords:** Nitrogen biobank, gene expression, congenital heart malformations, CVD, mRNA, DNA.

## Abstract

Biobanks play a crucial role in "-Omics" research providing well-annotated samples to study major diseases, their pathways and mechanisms. Accordingly, there are major efforts worldwide to professionalize biobanks in order to provide high quality preservation and storage of biological samples with potentially greater scientific impact. Biobanks are an important resource to elucidate relevant disease mechanisms as well as to improve the diagnosis, prognosis, and treatment of both pediatric and adult cardiovascular disease. High-quality biological sample collections housed in specialized bio-repositories are needed to discover new genetic factors and molecular mechanisms of congenital heart disease and inherited cardiomyopathies in order to prevent the potential risk of having a fatal cardiac condition as well as to facilitate rational drug design around molecular diseases (*personalized medicine*). Biological samples are also required to improve the understanding the environmental mechanisms of heart disease (*environmental cardiology*). The goal of this paper is to focus on preanalytical issues (informed consent, sample type, time of collection, temperature and processing procedure) related to collection of biological samples for research purposes. In addition, the paper provides an overview of the efforts made recently by our Institute in designing and implementing a high-security liquid nitrogen storage system (-196°C). We described the implementations of reliable preservation technologies and appropriate quality control (the right temperature, the right environment, fully traceable with all possible back-up systems) in order to ensure maximum security for personnel as well as the quality and suitability of the stored samples.

## INTRODUCTION

The establishment of biobanks around the world has become an integrated part of modern healthcare [1, 2]. In 2009, the potential of a biobank for research purposes has been acknowledged among 10 ideas that are changing the world [3], and at least 179 biobanks exist in the United States, most of which were established in the last 10 years (2). A Biobank may be defined as a “*system which will store one or many types of biologic specimens for later analysis from single or multiple studies which permit efficient retrieval and optimal stability of the samples*” [4].

Indeed, the storage of human samples (blood samples, isolated cells and fractions) represents a great opportunity for the medical science in order to discover biomarker and molecular signatures of most common diseases, such as cancer, cardiovascular diseases, neurodegenerative diseases, and diabetes.

Clinically validated biomarkers may provide information to be used for the risk/predisposition, diagnosis, screening, monitoring of prognosis and prediction of response to treatment [1-4].

Thus, there are currently major efforts worldwide in order to move from a “do-it-yourself” tissue collection- as is most frequent at present- to professionalized biobanks for providing high quality preservation and storage of biological samples with potentially greater scientific impact [5, 6]. 

Unfortunately, despite the importance and complexity of this issues, there is very little published information concerning the suitable methods for a correct preservation and storage of biological samples as well on the design and development of a biobank.

The aim of the paper is to give a view based on our recent experience in the design and development of a liquid nitrogen biobank as a key resource for clinical research.

## THE IMPORTANCE OF CRYOBANKED SAMPLES FOR “OMICS” STUDIES

In spite of decades of clinical and epidemiological research, the etiology of many complex (multi-factorial) diseases is still largely unknown.

The ‘omics’ technologies, including transcriptomics, proteomics, and metabolomics, are revolutionizing our understanding of mammalian biology, promising the discovery of new biomarkers and raising the perspective of “personalised medicine” [7-10].

Transcriptomics is the identification and characterization the mRNA transcripts that are present in a sample at the time of collection by using the tools of molecular biology and bioinformatics. Gene expression profiling can be performed by using high throughput techniques such as microarray technology in order to determine which genes are differently expressed as result of changes in diseased conditions or in environmental conditions [7-10].

Proteomics is the study of all proteins present in specific cell types or tissues in order to understand their role in pathophysiological conditions. In contrast to the genome, the proteome is highly dynamic over time, between cell types and in response to the environment [7-10].

Metabolomics is defined as the study of small metabolites involved in the energy transmission in cell by interacting with other biological molecules following metabolic pathways [7-10]. Metabolomic studies are performed in easily collected biological samples, such as urine, saliva or plasma, with nuclear magnetic resonance (MR) spectroscopy and mass spectrometry to identify the chemical components of biologic pathways (substrates, enzymes, and their products) produced by changes in gene and protein expression.

Furthermore, regulations of gene expression by epigenetic mechanisms has emerged as one of the most crucial determinants of cellular behaviour of common diseases [11, 12].

Indeed, epigenetics describes the mechanisms that enable cells to respond quickly to environmental changes and provide a link between genes and the environment [12]. 

Finally, the concept of the 'exposome'- reflecting the cumulative effect of exposure from the gestation onwards- has recently been introduced as a complement to the genome in studies of disease etiology [13, 14].

The exposome concept promotes the application of "-omics" tools in environmental health by comparing biological samples from diseased and healthy subjects in order to better characterize intermediate pathways, potentially providing the 'missing links' among exposures, genes, and diseases [15, 16].

In this light, the most important prerequisites for “omics” studies is the careful handling and storage of precious biological sample in order to preserve the quality of the final stored samples of most epidemiological and clinical studies.

**Fig. (1):**
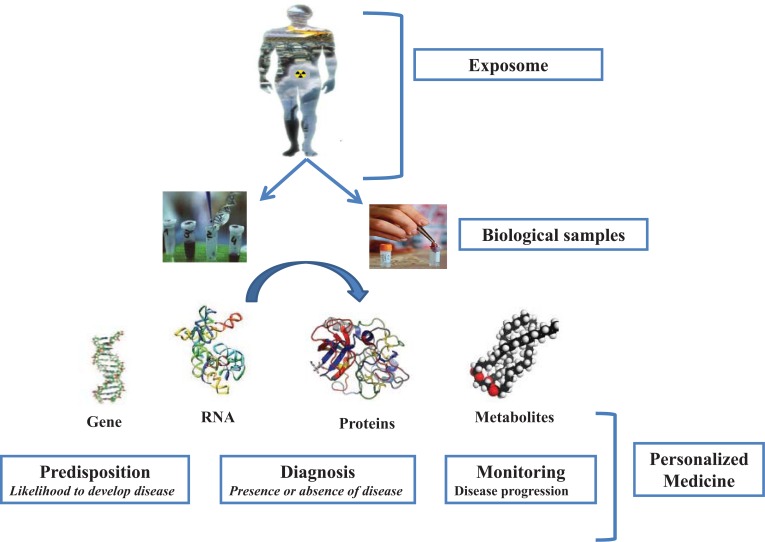
The importance of biomolecules for "-omics" studies in Environmental and Personalized Medicine.

The biomolecules that are currently of major value in modern biobanking, retained in biofluids and tissues, are DNA, mRNA, proteins, peptides, phospholipids, and small metabolites (Fig. **1**).

## BIOBANK FOR CARDIOVASCULAR DISEASE RESEARCH

Cardiovascular diseases (CVD) remains the leading cause of mortality and morbidity in both children and adults [17, 18]. Atherosclerotic heart disease and valve disease are serious health problem and leading causes of death in industrialized and developing countries [18].

Advances in cardiology research during the last years has allowed the discovery of numerous potential biomarkers on protein or even genetic level. These biomarkers are an exciting new tool for guiding diagnosis and treatment as well as for facilitating the individualization of adult patient care. Nevertheless, considerable uncertainty remains with regards to the incremental predictive value of a panel of biochemical and genetic biomarkers beyond conventional risk factors. Integrated approaches, combining circulating, imaging, and genetic biomarkers, are required to make a complete assessment of the potential clinical usefulness of biomarkers for risk prediction of CVD disease [19].

Congenital heart malformations are the most prevalent and fatal of all birth defects, occurring in nearly 1 in 100 live births [20]. Before the advent of cardiac surgery for congenital cardiac malformations, less than one fifth of children born with such lesions reached adulthood. The progress of surgical management, and more recently interventional catheterization, has allowed an increasing number of congenital heart defects to be corrected surgically so that an increasing number of patients reach adolescence and adult life, even those with the complex defects [21]. Despite these advancements in medical and surgical therapy, many questions still remain open about the disease, regarding the genetic and molecular bases of different forms of congenital heart defects [22, 23]. Future studies and more research in this area are greatly needed to provide insight into the molecular basis of disease as well as to answer these questions [22, 23].

Inherited cardiomyopathies, including ion channelopathies, are among the most common genetic cause of sudden cardiac death, especially in the young (including trained athletes). Important progress has recently been made in identifying the genetic causes of cardiomyopathies, which, in turn, has provided new opportunities for practitioners, patients, and families to use this genetic information [24]. However, the genetic cause has been identified for only a portion of these life-threatening genetic disorders: up to 75% of the long-QT syndrome, 65% of hypertrophic cardiomyopathy. 50% of arrhythmogenic right ventricular cardiomyopathy/dysplasia and catecholaminergic polymorphic ventricular tachycardia, and 25% to 30% of dilated cardiomyopathy and Brugada syndrome [25].

Further research is, therefore, needed to discover new mutations and understand the role of the genetic factors underlying variable penetrance and variable expressivity as well as the function of genetic modifiers, common polymorphisms, multiple mutations, and epigenetic mechanisms. 

Specifically, in cases where no definitive cause is identified at postmortem (i.e. Sudden Unexpected Death), the “molecular autopsy” may be as a key process in the investigation of the cause of death [26]. Furthermore, there is now compelling evidence that environmental factors can dramatically impact on the development of CVD. In addition to tobacco smoke, certain carcinogenic environmental agents, including air pollution, arsenic, dioxin and ionizing radiation exposures, have been described to elevate the risk of cardiovascular disease, ischemic heart disease, arrhythmias, and heart failure [27] and congenital heart disease [22].

In spite of the epidemiological evidence indicating that environmental toxins can heart disease, the mechanisms of the cardiovascular injury and heart effects are not fully understood. 

Clearly, additional studies are required in order to improve the understanding of environmental mechanisms of heart disease but also in developing specific preventive or therapeutic strategies for minimizing the environmental mechanisms of heart disease. 

Therefore, biobanks are an important resource for cardiovascular research to elucidate relevant disease mechanisms as well as to improve the diagnosis, prognosis, and treatment of CVD diseases. Indeed, storage of blood and tissue samples (through heart transplant, heart surgery, biopsy or autopsy) from diagnosed CVD patients and from experimental models is of great importance to identify pathways involved in disease initiation or progression in order to prevent the potential risk of having a fatal cardiac condition as well as to facilitate rational drug design around molecular diseases (personalized medicine). 

## DESIGN OF SAMPLE COLLECTION FOR HIGH QUALITY BIOBANKING

When building a long-term archive of biological material for future research, many factors must be considered at the initial collection stage, and it is, therefore, recommended the strict adherence to standardized protocol in order to obtain high quality biobanks [28-30]. The workflow of the main aspects of the biobanking process is outlined in Fig. (**2**).

Besides methodological issues, a crucial prerequisite to collect samples for a biobank is the ethical approval from an Institutional Review Board or Institutional Ethics Committee [5].

In accordance with applicable law and ethical principles pertaining to the protection of human subjects (the UNESCO *Universal Declaration on Bioethics and Human Rights *(2005), UNESCO *Universal Declaration on the Human Genome and Human Rights *(1997), *Declaration of Helsinki on Ethical Principles for Medical Research Involving Human Subjects *(1964, last revised 2008), biological samples should be acquired only with the informed consent of participants, who, thus, transfer for free their powers on the biological samples to the biobank, also defining the limits within which they can be used [5, 29].

In some circumstances, where authorised by applicable law and the appropriate authorities, a relatively broader consent may be obtained to enable the use of human biological specimens to address unforeseen research questions at time of collection, as new biomarkers become available [5, 29]. 

For instance, according to the Italian Authority for the Protection of Personal Data: “The storage and further use of biological samples and genetic data collected for research projects and statistical studies, different from those for which the informed consent was originally acquired, are permitted but limited to the pursuit of scientific and statistical purposes directly related to the original aims” [29].

Anyway, patients may request in writing at any time the possibility for withdrawing consent including the destruction of biological samples and data [5, 29]. 

As regard the sample collection process, the quality of the operating methodologies with respect to processing and storage is extremely important for getting the desired quality of samples. 

When designing research studies with biological sample, researchers are faced with the question of how to store a vast number of samples in a way that allows the greatest number of analytical options in the future [30-32]. 

A general recommendation is that the protocol for the collection, processing and archiving of biological samples should include as many sample types as possible in order to provide a resource for a wide range of future scientific questions [30-32].

Different technical aspects apply to collection of different types of samples, such as the careful selection of anticoagulants and preservatives in blood collection tubes, the temperature and length of storage prior to processing in order to avoid loss or damage [30-31].

Plasma is collected in a tube with anticoagulant added (commonly EDTA, citrate, or heparin), whereas serum is collected into a tube that contains no additives or in many cases a form of micronized silica to promote clotting. For genetic analysis, the collection of EDTA-blood is good for DNA-based assay. 

Furthermore, time is another crucial factor throughout the process of sample collection and processing as *ex vivo* cellular injury, disintegration and cellular granule release, and action of proteases may occur after sampling. Indeed for some types of biomarkers it might be feasible to add a mixture of protease inhibitors in order to avoid extensive degradation of the proteins in the sample.

Although blood samples are often kept at room temperature until processed, a general recommendation is that the sample is separated as rapid as possible into different components (plasma, cells) and each component is kept at the appropriate temperature [30-31]. The sooner the separation of plasma from cells occurs, the higher the quality of the resultant sample. This is particularly important for biomarkers that are both circulating and stored in platelets [33].

When the samples are not being analysed immediately, they should be maintained at low temperature because room temperature results in degradation of labile protein biomarkers (e.g. cytokines), anti-oxidants (ascorbic acid, uric acid, a-tocopherol), and other analytes (such as folate and vitamin B12).

Indeed, temperature control during the processing of samples and the long-term storage is essential and determines the usefulness of samples in future analyses [34-36].

As general recommendation, storage at −80 °C over a prolonged time is adopted as a minimum for aliquots of serum and plasma which contain a large number of soluble molecules most require very low temperature to remain intact [33-35]. Anyway, some biomarkers may degrade spontaneously during storage even at −80 °C and storage in liquid nitrogen) may be required for these biomarkers [37]. Conversely, isolated DNA can be stored at 4 °C for several weeks, at −20 °C for several months, at −80 °C for several years. Isolated RNA must be stored at −80 °C [38]. Live cells are stable at room temperature for up to 48 h but must be either cultured or cryopreserved in liquid nitrogen in order to remain alive [39].

## LONG-TERM SAMPLE STORAGE: WHY CHOOSE A LIQUID NITROGEN BIOBANK?

The best method for preserving cells and biomolecules is their cryopreservation in liquid nitrogen which is becoming an increasingly common storage option for biomedical research [40-42]. The objective of cryopreservation is to minimize damage to biological materials. The lower the temperature, the longer the viable storage period. The term ‘cryopreservation’ (cryogenic preservation) refers to the storage of cells, tissues and organs following appropriate “preparatory” procedures at the ultra-low temperature of liquid nitrogen (-196°C). At this temperature, the vegetative cells enters in a state of “absolute quiescence”, as all the physical and thermally driven reactions are practically halted; in this particular condition, it is believed that the majority of stored cells will remain viable and unchanged for an indefinite period [40-42].

However, the establishment and maintenance of liquid nitrogen biobanks require careful planning to ensure that personnel using the facility are working in safe condition and the samples are maintained in a suitable environment for long-term storage by using reliable preservation technologies and appropriate quality control.

The most important safety consideration is the potential risk of asphyxiation when escaped nitrogen vapourises and displaces atmospheric oxygen, leading to oxygen depletion which, in turn, can very rapidly cause loss of consciousness. Consequently, liquid nitrogen refrigerators should be subject to periodically preventative maintenance and should have low oxygen alarm systems.

In addition, it is important that staff are trained in the use of liquid nitrogen and individual protective equipments in order to minimize the risk of adverse incidents. Table **1** summarizes the precautions for liquid nitrogen storage banks.

Accordingly, our Institute at CNR Campus (Pisa, has recently chosen to set up a high-security liquid nitrogen storage system in order to provide the highest security and quality standards to long-term storage (years to decades) for biologic specimen collections from current and previously funded projects.

The core of the liquid nitrogen facility is an appropriate room containing the cryo-containers connected to a fixed external station for periodically refilling of liquid nitrogen.

Access to biobank is controlled though electronic bagdes and all the processes are managed by a specially designed software system (Sample Management and Real Traceability:CryoSMART, Air Liquide Cryogenic Equipments Division, Italy) that automatically and securely manages the supervision and the surveillance of cryobiological storage samples, including traceability of the storage conditions.

Indeed, this system allows users to track biosamples from acquisition to analysis, to store annotations associated with those samples as well as to constantly monitor parameters and critical operating conditions such as oxygen level, management of the supply in liquid nitrogen, synchronization of the fillings of cryo-containers and management of the acoustics and visual alarms from environmental sensors. Any alarm that is identified as “critical” will be remotely sent by SMS, vocal announcement or by e-mail to operators in order to allow intervention in case of emergencies Fig. (**3**).

This facility can ultimately house 25 vapor-phase liquid nitrogen cryo-containers with a storage capacity of thousands or even millions of unique biological specimens providing a more professional service of biobank samples and processes. 

## ADVANTAGES, COSTS AND PITFALLS OF NITROGEN BIOBANK 

Liquid nitrogen biobank has gained popularity as refrigeration option of storage for biomedical research also for significant reductions in maintenance time and cost as compared to mechanical freezers. Indeed, cryogenic storage using liquid nitrogen is the most effective long-term storage platform because the extreme cold slows most chemical and physical reactions of the more complex biological structures. In addition, nitrogen cooled systems requires low electricity usage, and thus, there are significant operating cost savings from the lower power requirements. Typically, a liquid nitrogen system has also between one and two orders of magnitude fewer working mechanical/electrical parts than other refrigeration systems. This greatly simplifies spares holding, reduces the risk of obsolescence and generally makes the system much easier to support over the long term. In a mechanical freezers samples are accessed by opening a large door, resulting in the inflow of warm air and moisture. For this reason, due to the risk of damage to samples, access times are limited and the time between placements or withdrawals must be carefully managed to ensure that the freezer contents can recover to -80°C. Accordingly, many of the larger biobanks are buying sophisticated and very expensive freezers to maintain a constant temperature [43]. Rather than opening freezer doors, researchers place sample tubes in a deliver hatch and a mechanical arm then moves them to interior shelves. The samples are deposited and an e-mail is sent when they are ready to be picked up [43]. In addition, cold refrigeration/freezers produce hydrofluorocarbons, which are some of the most potent greenhouse gas pollutants with a deleterious impact on the environment. It has been estimated that the typical ultra-low-temperature freezer consumes about 7,665 kWh per year while releasing 54,805 pounds of carbon dioxide. This is equal to the emission from about four cars [44]. Conversely, nitrogen is common in the air we breathe every day. There are thus no adverse environmental effects from discharging the nitrogen gas into our environment Therefore, use of nitrogen is an eco-friendly and highly sustainable process, yielding many performance advantages as greater sample stability, lower temperature and flexibility in refrigeration loading, low-capital requirement, significant reductions in maintenance time and cost. According to the scopus, biobanks can vary tremendously in size, scope and focus. Samples can be collected from the general population or from patients solely for research purposes. However, infrastructure investment and operating cost requirements even for a modest biobank is a significant commitment and requires adequate financial support. The initial investment costs are needed to install the instrumentation as well as dedicated personnel, including several key individuals dedicated exclusively to the activities of the biobank in order to guarantee the quality control and technical duties, such as biomaterials collection and database organization. An appropriate space for the liquid nitrogen tanks with an automatic refilling system is required with controlled temperature and ventilation, and monitoring of the partial pressure of oxygen. The costs of a liquid nitrogen tank is based on the size and capacity of the instrument and can vary from 19,500 to 52,000 $ (15.000-40.000€). The cost of liquid nitrogen must be also added. The daily cost of liquid nitrogen varies depending on the volume of the tanks and is about 30,000/year $ per 1000 samples (23.000€). To calculate annual personnel costs, at least 2 a full-time laboratory technicians supported by two-staff scientists are necessary. In addition, it is estimated that the director would dedicate about 30-50% of his/ time in activities relative to the biobank. Sustained funding and support is also needed for the long-term maintenance of biobank in order to adequately guarantee its functionality.

## CONCLUSION

The introduction of"-omic" high-throughput technologies in cardiovascular research opens unprecedented opportunities for improving the understanding of complex (multi-factorial) diseases. Biobanks are a critical resource for such molecular-based biomedical research, playing a key role in the area of personalized medicine in which the treatment will no longer be “one size fits all”, but instead “tailored” to the molecular and genetic profile of each patient. Despite this potential, researchers often overlook the critical points which can affect the quality of biological samples. Therefore, we would highly encourage Researcher Centers to support and develop well-organized Biobanks which include a high degree of expertise in issues related to sample collection and processing, and implementations of reliable preservation technologies and appropriate quality control in order to ensure the quality and suitability of the stored samples. 

This strategy will offer to identify and validate new diagnostic and therapeutic approaches based on novel biomarkers and molecular targets, promoting their translation into clinical practice.

## Figures and Tables

**Fig. (2):**
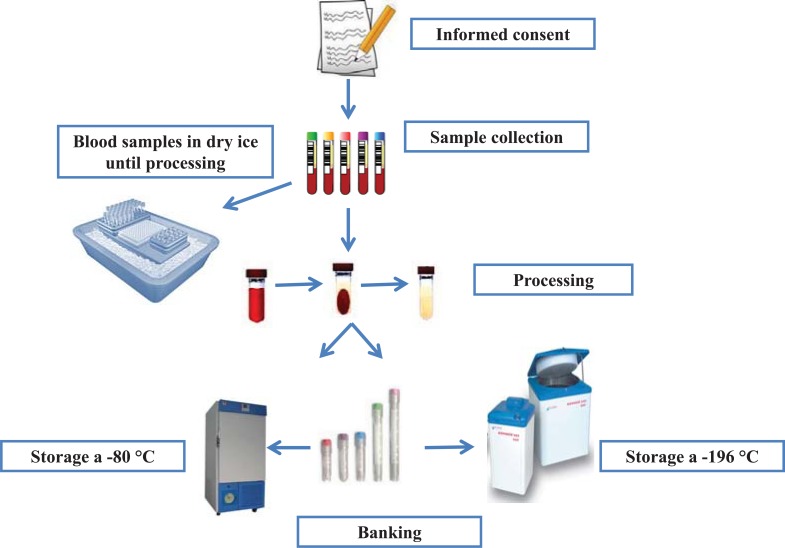
Schematic representation of the biobanking process.

**Fig. (3):**
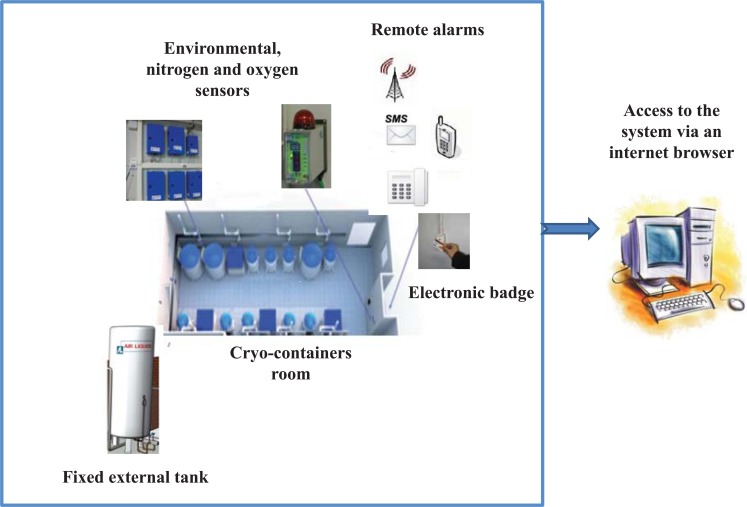
Schematic model of a Biobank structure and organizational control system.

**Table 1.** Precautions for Liquid Nitrogen Biobank

Access restricted to authorized staff
Staff training (work in pairs)
Use of personal protective equipment (full-face visor; thermally insulated gloves, apron)
Mechanical ventilation systems
Oxygen alarms set to 18% oxygen (v/v)
Immediately leave the room if subject begin to experience any symptoms of oxygen deficiency (dizziness; deeing spots; rapid breathing; poor coordination)
